# Epidemiological profile of female firearm-related mortality

**DOI:** 10.1097/MD.0000000000024222

**Published:** 2021-01-15

**Authors:** Dayane Caroliny Pereira Justino, Ketyllem Tayanne da Silva Costa, Fábia Barbosa de Andrade

**Affiliations:** a Master in Collective Health, Programa de Pós-Graduação em Saúde Coletiva; b Programa de Graduação em Enfermagem, CNPq (Conselho Nacional de Desenvolvimento Científico e Tecnológico; c Doctor of Health Sciences, Departamento de Enfermagem, Federal University of Rio Grande do Norte, Natal, Brazil.

**Keywords:** external causes, mortality, violence against women, women's health

## Abstract

The mortality rate of women due to firearms increases every day in Brazil and globally. This study aimed to evaluate the trends of firearm-related mortality in women from the years 2007 to 2016 in order to determine their profile and to associate these indicators with public policy and strategies to reduce mortality.

This is an ecological time-series study using secondary data of women aged 10 to 49 years old collected through the mortality information system (SIM) in Brazil. Furthermore, independent characteristics such as education, color, race and civil status were also collected from SIM. Data was analyzed using the Join Point open source software version.

There was an increase in the mortality rate of women who received 4 to the 7 years of education, were single, and brown-skinned. There was a significantly increased rate of mortality in women whose ages ranged from 20 to 29 years followed by 30 to 39 years; the rate was also significantly higher in the northeast region followed by the southeast region.

There is a need for professional training to assist women in vulnerable situations.

## 1 Introduction

In Brazil, external causes of death, referring to events which cause injury and adverse effects on the body such as accidents and interpersonal violence, are configured as a major public health problem, and these are directly reflected by public health spending on hospitalizations.^[1]^ A recent Pakistani study demonstrated that most women who suffered intimate partner violence did not report the incident and did not seek counseling services.^[2]^

Women aged 10 to 49 years are referred to as Women of Childbearing Age (FIM), meaning this is the period wherein women usually conceive and start a family.^[3]^ The FIM is a Brazilian concept that is used to improve public health services, and this study sought to study women in this age group considering the social importance of women of reproductive age, since high mortality rates in this group reflect a problem in public strategies and policies aimed at womens health in addition to impacting the structural reduction of birth rates and active adults in society.

Stöckl^[4]^ found that approximately 40% of homicides of women in the world are committed by their partners and that homicides are usually accompanied by a history of abuse, whether physical, sexual, and/or psychological. A study carried out in Chicago in 2011 shows that the young women aged 20 to 29 years who died due to firearm-related homicide had characteristics which made them highly vulnerable, such as partaking in drug use and trafficking, alcohol use, and gang membership, and they died at a rate 5 times higher than women in the general population.^[5]^

The deaths of women due to external causes has risen at a significantly statistical rate over the last decade (2000–2010) in many countries, being the third most common cause of death in Brazil, mainly due to physical aggression.^[6]^ Although firearm-related deaths occur at a greater rate in men, there has been an increase in this type of death among women in Brazil in recent years.^[7]^ In a study carried out in the state of Acre, majority of the women who died due to firearm-related causes from 2002 to 2010 were aged between 21 and 25 years, non-white, had a low level of education, no occupation, and resided in an urban area.^[8]^

Local studies are important in investigating local realities, in organizing and planning carried out by the manager, and contribute to the formulation and adjustment of public health policies. It is necessary to study what happened to these women, to identify the scenarios of these women, and make it known how serious this is as a public health problem, in addition to building strategies and plans for protecting women.^[9]^

In view of the complex problem of violence against women, the United Nations (UN) says that it is essential to ensure that women in situations of violence have access to a series of essential services provided by different sectors, such as health, the police, justice, and social services. These services are aimed at mitigating violence against women and reducing their effects on the well-being, health, and safety of victims, as well as aiding in their recovery.^[10]^

In Brazil, a law called Maria da Penha was instituted in 2006 with the aim of eliminating all forms of discrimination against women and eradicating violence against women, not only involving physical violence and aggression, but also psychological violence.^[11]^

Given this information, this study aimed to evaluate the trends of firearm-related mortality in females from 2007 to 2016, to identify their profile, and to associate these with public policies and strategies used in Brazil.

## 2 Material and methods

This is an ecological time-series study which was carried out in Brazil using secondary data. The study included women aged 10 to 49 years old. Brazil has 5570 municipalities and it is divided in 5 regions – North, Northeast, Midwest, South, and Southeast.

In Brazil, women aged 10 to 49 years old are considered to be of childbearing age, and this definition was based on studies of vital records and medical procedures that showed that at this stage, women are sexually active and have a higher chance of reproducting.^[12]^ In light of this information, the Pan American Health Organization (PAHO) says that external causes such as accidents and violence constitute a serious public health problem.^[13]^

The data were collected from the Informatics Department of the Unified Health System (DATASUS),^[14]^ specifically from the Mortality Information System (SIM) from the years 2007 to 2016. The data were chosen based on the type of firearm aggression, specifically assault firing using a firearm, assault firing of a larger caliber, and assault firing using another firearm, or unspecified.

The dependent variable of the study was the female firearm-related mortality rate from 2007 to 2016, which was calculated by dividing the number of firearm-related deaths in women aged between 10 and 49 years over the period of 2007 to 2016 by the resident population of women of the same age based on a census conducted in 2010 by the Brazilian Institute of Geography and Statistics (IBGE),^[15]^ multiplied by 10,000. The independent variables which included education, color, race, and civil status were also collected from SIM.

After data collection, data were processed and stored in the Microsoft Excel Software, where the database was cleaned, replacing terms of “-“ or “.” with “0,” and the relative frequency (%) was calculated. The Join Point open source software version 4.7.0.0 (Surveillance Research, National Cancer Institute, USA) was used to analyze trends using junction point models, to conduct a historical series through Poinsson regression, to estimate the Annual Percentage Change (APC) and the Average Annual Percentage Change (AAPC) of a segmented linear regression, and to identify inflection points that reflect changes in the increase or decrease in the rates. There was no need for an appraisal by the Ethics and Research Committee, considering that public domain data were used in this study.

## 3 Results

The characteristics of the female victims of firearms are described in Table 1, showing that the highest occurrence of these deaths is in women who received 4 to 7 years of schooling, were single, and brown-skinned.

**Table 1:** Percentage of education, civil status and race of victims of firearms in Brazil from 2007 to 2016 (2019).

	10 to 19 years	20 to 29 years	30 to 39 years	40 to 49 years	Total
	N (%)	N (%)	N (%)	N (%)	N (%)
YEARS OF EDUCATION^∗^					
Not study	24 (80)	81 (270)	101 (336)	94 (313)	300 (100)
1 to 3 years	528 (214)	783 (318)	717 (291)	434 (176)	2462 (100)
4 to 7 years	1.893 (289)	2354 (359)	1538 (234)	765 (116)	6550 (100)
8 to 11 years	813 (193)	1798 (4279)	1056 (251)	535 (127)	4202 (100)
12 years or more	58 (61)	386 (411)	258 (304)	208 (221)	937 (100)
CIVIL STATUS					
Single	3.999 (275)	5.872 (404)	3316 (225)	1325 (91)	14512 (100)
Married	52 (25)	466 (225)	813 (395)	740 (357)	2071 (100)
Widow	4 (16)	31 (125)	80 (323)	12 (534)	247 (100)
Divorced	8 (13)	91 (149)	238 (392)	270 (444)	607 (100)
COLOR/RACE					
White	1214 (197)	2181 (354)	1659 (269)	1098 (178)	6152 (100)
Black	281 (216)	468 (361)	362 (279)	184 (142)	1295 (100)
Yellow	3 (130)	6 (260)	5 (217)	9 (391)	23 (100)
Brown	2691 (239)	4291 (382)	2854 (254)	1381 (123)	11217 (100)
Indian	4 (143)	14 (500)	7 (250)	3 (107)	28 (100)

∗ years of study.

A regression analysis of the firearm-related mortality rate of women by age group was also performed (Table 2). Data analysis using Join Point identified a pattern of occurrence. When the statistical significance of the regression through the points of junctions was verified, it was found that the rates were significantly higher for women aged 10 to 19 years old in the north, northeast, and southeast regions, while the rates were also significantly higher in women aged 20 to 29 years in the north and northeast. The rates in women aged 30 to 39 years old were significantly higher in the northeast and south, and those in women aged 40 to 49 years old were significantly higher in the north. Table 2 shows that the highest average occurrence of firearm-related mortality occurred in women aged 20 to 29 years followed by 30 to 39 years, and this was also highest in the northeast region followed by the southeast region.

**Table 2:** Annual variations of trends in the female firearm-related mortality rate in Brazil and by region from 2007 to 2016 (2019).

Local	Age group	Average^1^	Jump Point	Period	APC^2^	Lower	Upper	AAPC^3^	Lower	Upper
Brazil	10–19	441.5	2011	2007–2012	5.0^∗^	0.5	9.7	−1.5	−3.2	−0.2
				2012–2016	−2.4	−8.1	3.6			
	20–29	732.3	2011	2007–2013	3.9^∗^	1.0	6.8	1.6	−1.0	4.1
				2013–2016	−2.9	−10.5	5.2			
	30–39	514.6	2011	2007–2012	6.7^∗^	3.2	10.4	3.4^∗^	1.3	5.6
				2012–2016	−0.6	−4.9	3.9			
	40–49	280.6	2011	2007–2013	2.3^∗^	0.1	4.6	4.0^∗^	2.5	5.5
				2013–2016	5.6	−0.4	12.0			
North	10–19	38.5	2011	2007–2012	22.4^∗^	8.2	38.6	11.8^∗^	3.9	20.3
				2012–2016	−0.2	−14.2	16.0			
	20–29	65.9	2011	2007–2012	15.5^∗^	6.9	24.7	9.2^∗^	6.7	11.8
				2012–2016	7.1	−1.7	16.7			
	30–39	46.4	2011	2007–2013	17.1	13.2	21.2	7.8^∗^	4.4	11.4
				2013–2016	1.2	−6.4	9.5			
	40–49	25.9	2011	2007–2013	4.7	−4.4	14.7	15.5^∗^	10.1	21.1
				2013–2016	24.1^∗^	3.4	49.0			
Northeast	10–19	163.7	2011	2007–2012	13.9^∗^	4.8	23.8	5.9^∗^	0.8	11.2
				2012–2016	−3.3	12.5	6.9			
	20–29	268.9	2011	2007–2013	8.1^∗^	2.1	14.5	4.9^∗^	0.1	10.1
				2013–2016	−1.1	−14.6	14.6			
	30–39	181.2	2011	2007–2011	15.1^∗^	7.0	23.7	6.4^∗^	3.2	9.7
				2011–2016	−0.1	−4.3	4.3			
	40–49	89.0	2011	2007–2010	12.8	−16.3	52.0	4.2^∗^	0.7	7.9
				2010–2016	2.6	−5.3	11.2			
Midwest	10–19	42.7	2011	2007–2010	−16.0	−33.3	5.7	−4.5	−10.9	2.4
				2010–2016	1.8	−5.4	9.7			
	20–29	70.0	2011	2007–2012	11.9	−1.3	26.7	5.5^∗^	1.1.	10.0
				2012–2016	−1.6	−15.9	15.1			
	30–39	48.6	2011	2007–2009	16.4	−50.4	173.6	−2.1	−16.4	14.6
				2009–2016	−6.8	−16.0	3.3			
	40–49	26.4	2011	2007–2012	6.1	−5.7	19.4	3.1	7.0	14.3
				2012–2016	−2.3	−30.0	35.1			
Southeast	10–19	134.8	2011	2007–2013	−12.9	−38.9	24.8	−6.5^∗^	−9.1	−3.9
				2013–2016	−5.2^∗^	−10.1	−0.1			
	20–29	229.7	2011	2007–2013	−3.7	−7.2	0.0	−6.9^∗^	−8.7	−5.0
				2013–2016	−11.1	−22.5	2.1			
	30–39	167.2	2011	2007–2011	−4.1	−8.7	0.7	−1.8^∗^	−2.9	−0.7
				2011–2016	−1.0	−4.6	2.8			
	40–49	93.9	2011	2007–2009	−11.1	−44.5	42.4	−1.7	−10.1	7.5
				2009–2016	1.2	−5.5	8.3			
South	10–19	61.8	2011	2007–2009	6.9	−34.4	72.1	−0.9	−9.6	8.6
				2009–2016	−2.9	−9.5	4.3			
	20–29	97.8	2011	2007–2009	25.4	−21.4	100.2	4.4	−4.2	13.8
				2009–2016	−0.9	−6.5	5.0			
	30–39	71.2	2011	2007–2012	20.3^∗^	3.0	40.6	7.1^∗^	3.2	11.2
				2012–2016	2.4	−14.9	23.2			
	40–49	45.4	2011	2007–2010	28.3	−52.4	245.9	8.3^∗^	2.2	14.8
				2010–2016	5.7	−5.4	18.0			

^1^mean number of cases during the studied period; ^2^annual percentage change; ^3^average annual percentage change.

∗ *P* value <.05.

**Figure 1:**
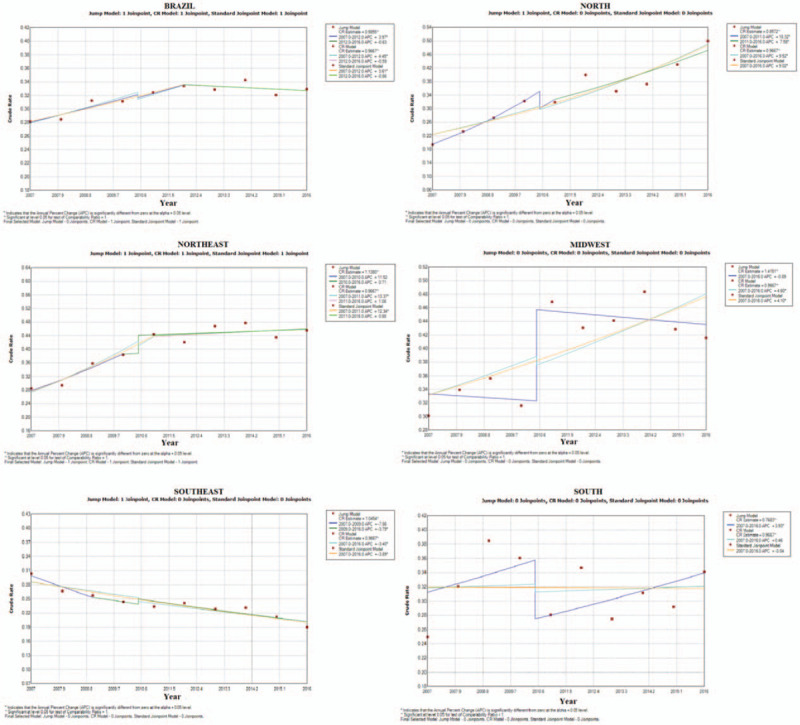
Polynomial regression with annual variations in female-firearm related mortality in Brazil and by region from 2007 to 2016 (2019).

Evaluating the regressions by region using the polynomial model (Fig. 1) showed that there is an expressive and significant increase in firearm-related mortality in women in the north, midwest, northeast, and south regions. There was also a statistically significant reduction in the southeast region over the studied period. Upon analyzing the curve of firearm-related mortality in women in Brazil, the mortality rate of female fire-arm related mortality increases until the year 2012 with statistical significance in the regression analysis; however, the reduction that occurs in the period from 2012 to 2016 did not show statistical significance.

## 4 Discussion

External causes have been the main cause of death in young Brazilians, the main causes attributed to urban violence where the aggressors are mainly young men.^[16]^ Certain risk factors predispose women to violence, such as having brown skin, being single, and having received little education (from 4 to 7 years), as shown in Table 1.

There is an increase in the mortality rate of women who received 4 to 7 years of education, single, and brown. Upon evaluating the average occurrence, it was found to be highest in women aged 20 to 29 years followed by those aged 30 to 39 years, and significantly higher in the northeast region followed by the southeast region.

This information, along with continuous surveillance, may aid in the continuous improvement of the information system. This enables managers, policymakers, and health care providers at various levels of the health system to identify deaths and to collect and analyze data, and it also helps strengthen the decision-making process in formulating recommendations and actions that will improve health outcomes.^[17]^

With regard to the educational attainment, the inequality in the average years studied decreased over the years. The average number of years studied is 6 times greater in the south eastern region as compared to the north eastern region, while the difference between the south and north eastern regions is almost imperceptible.^[18]^ However, it is important to highlight that the north eastern region has the highest average occurrence of female firearm-related mortality, while the north has the lowest average occurrence.

A study by Barufaldi et al^[19]^ carried out from 2011 to 2015 in Brazil shows that the female victims of violence were mostly black and had less than 7 years of schooling. While in our study, majority of female victims were brown-skinned, the number of years of schooling is similar.

A study by Reichenheim et al^[20]^ revealed that in addition to regional differences, violence in Brazil is also influenced by socio-cultural determinants, being associated with the misuse of alcohol and other drugs and the wide availability of firearms.

Some of the factors which influence homicide in Brazil are inequality and poverty.^[21]^ Taking this perspective, a study conducted in north eastern Brazil identified a direct relationship between the mortality rate and the socioeconomic condition and an inverse relationship between the spending public and health, showing that these had a direct influence on the population's quality of life.^[22]^

Regarding income, the findings show that the greatest occurrence of female firearm-related deaths were concentrated in 2 regions which had the greatest socio-cultural inequality; one of them ranks second in the country poverty indicator. Studies show that the homicide rate had decreased by 39.0% in middle-income countries and 10.0% in low-income countries. However, the risk of death in Brazil is 10 times higher as compared to countries with a high income.^[20,21]^

Upon investigating the Potential Years of Life Lost (APVP) of these women, there was an average of 16 years of life lost, even after the implementation of the network of confrontation for women victims of violence which established womens police stations, shelter houses, centers of reference, and even the Maria da Penha Law.^[11]^ This shows that given the average years of life lost in Brazil, these women are still highly vulnerable. Enacting public health policies for female victims of violence, such as a risk-assessment of women, may contribute to reducing these deaths in and in preventing new mortalities.^[23]^

Based on the technical notes and policies for preventing violence against women, there are actions executed from the first episode of aggression, in addition to preventing the consequences of violence suffered by women who found life after being raped.^[23]^

A study carried out by professionals specializing in Primary Care in the districts of Ribeirão Preto, São Paulo state in 2012 regarding the identification of women vulnerable to aggression that could result in death showed favorable results about how to identify victims and where to direct women in this situation.^[24]^ Although this is a local study, it explains the reduction in female firearm-related mortality presented in the southeast region in Figure 1.

Thus, training professionals working in Primary Care proved to be effective and is of fundamental importance in identifying women in situations of aggression and may consequently reduce mortality.

## 5 Conclusions

The results showed that since 2007, the rate of firearm-related deaths in women has been increasing and this places a significant burden on the population. Furthermore, the indiscriminate use of firearms affects various people and systems associated with the victim, such as family members, health professionals, and the financial sector.

This study is socially relevant in that it considers the health of young women and the attention that is provided to them by the health service, in addition to revealing regions where greater attention is needed in terms of policies to prevent firearm-related mortality. There is a need for professional training in assisting these women in order to identify what factors make them vulnerable in order to prevent further mortalities. It is also necessary to establish a health care network for women victims of aggression as this can make a positive impact and minimize mortality.

This study has limitations because it is an ecological study using secondary data; therefore further research is necessary regarding women and their health situation in order to improve laws and public policies.

## Author contributions

**Conceptualization:** Dayane Caroliny Pereira Justino, Ketyllem Tayanne da Silva Costa, Fábia Barbosa de Andrade.

**Data curation:** Dayane Caroliny Pereira Justino, Ketyllem Tayanne da Silva Costa, Fábia Barbosa de Andrade.

**Formal analysis:** Dayane Caroliny Pereira Justino, Ketyllem Tayanne da Silva Costa, Fábia Barbosa de Andrade.

**Funding acquisition:** Dayane Caroliny Pereira Justino, Ketyllem Tayanne da Silva Costa, Fábia Barbosa de Andrade.

**Investigation:** Dayane Caroliny Pereira Justino, Ketyllem Tayanne da Silva Costa, Fábia Barbosa de Andrade.

**Methodology:** Dayane Caroliny Pereira Justino, Ketyllem Tayanne da Silva Costa, Fábia Barbosa de Andrade.

**Project administration:** Dayane Caroliny Pereira Justino, Ketyllem Tayanne da Silva Costa, Fábia Barbosa de Andrade.

**Resources:** Dayane Caroliny Pereira Justino, Ketyllem Tayanne da Silva Costa, Fábia Barbosa de Andrade.

**Software:** Dayane Caroliny Pereira Justino, Ketyllem Tayanne da Silva Costa, Fábia Barbosa de Andrade.

**Supervision:** Dayane Caroliny Pereira Justino, Ketyllem Tayanne da Silva Costa, Fábia Barbosa de Andrade.

**Validation:** Dayane Caroliny Pereira Justino, Ketyllem Tayanne da Silva Costa, Fábia Barbosa de Andrade.

**Visualization:** Dayane Caroliny Pereira Justino, Ketyllem Tayanne da Silva Costa, Fábia Barbosa de Andrade.

**Writing – original draft:** Dayane Caroliny Pereira Justino, Ketyllem Tayanne da Silva Costa, Fábia Barbosa de Andrade.

**Writing – review & editing:** Dayane Caroliny Pereira Justino, Ketyllem Tayanne da Silva Costa, Fábia Barbosa de Andrade.

## Abbreviations

Abbreviations: AAPC = Average Annual Percentage Change, APC = Annual Percentage Change, APVP = Potential Years of Life Lost, DATASUS = Informatics Department of the Unified Health System, FIM = Women of Childbearing Age, IBGE = Brazilian Institute of Geography and Statistics, SIM = Mortality Information System, USA = United State of America.
